# In-gel proteasome assay to determine the activity, amount, and composition of proteasome complexes from mammalian cells or tissues

**DOI:** 10.1016/j.xpro.2021.100526

**Published:** 2021-05-04

**Authors:** Ayse Seda Yazgili, Thomas Meul, Vanessa Welk, Nora Semren, Ilona E. Kammerl, Silke Meiners

**Affiliations:** 1 Comprehensive Pneumology Center (CPC), University Hospital of the Ludwig-Maximilians-University (LMU) and Helmholtz Zentrum München, Member of the German Center for Lung Research (DZL), Max-Lebsche Platz 31, 81377 Munich, Germany

**Keywords:** Antibody, Cell Biology, Molecular Biology, Protein Biochemistry

## Abstract

This protocol describes an easy and reliable in-gel proteasome assay to quantify the activity and composition of different proteasome complexes in cells and tissues. The assay works well with limited amounts of total cell protein lysates. Although this assay is optimized specifically for the proteasome chymotrypsin-like activity, it can be expanded to other proteasome activities as well. Using antibodies that detect distinct proteasome subunits or regulators, we can determine the composition and relative quantity of active proteasome complexes.

For complete details on the use and execution of this protocol, please refer to Meul et al. (2020).

## Highlights

• Maintain the activity of proteasome complexes by nondenaturing conditions
• Quantify the specific activities of each proteasome complex
• Determine the subunits and the bound regulators of proteasome complexes
• Analyze the relative distribution of proteasome complexes upon treatments

## Graphical abstract

**Figure:**
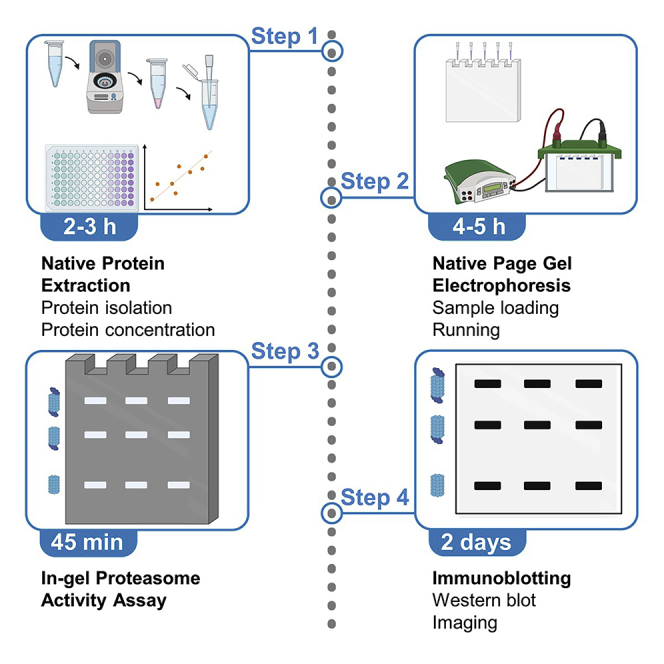

## Before you begin

### Native protein extraction

**Timing: 2–3 h**

1. To maintain active and assembled proteasome complexes, protein isolation should be done using non-denaturing buffer conditions. For this reason, we either use TSDG (TriS, DTT, Glycerol) buffer or OK (OverKleeft) Lysis Buffer containing a protease inhibitor cocktail. These two lysis buffers do not affect the outcome of the assay (Figure 1). However, we encourage you to stick to one of the lysis conditions for different experiments.
  a. Sample lysis with TSDG Lysis Buffer requires liquid nitrogen. After the cell or tissue pellet has been dissolved completely in TSDG buffer in safe-lock tubes, the tube is immediately placed carefully in liquid nitrogen. Continue doing this for all your samples to be lysed. Once all the samples are frozen in liquid nitrogen, transfer the samples to water (at 20°C–23°C) and keep them there until the buffer is completely thawed. Once the buffer is defrosted, transfer the samples back to the liquid nitrogen. Repeat this freeze and thaw cycle seven times. After the seventh thawing process, centrifuge the samples at 4°C for 20 min at 27,670 × *g.* Supernatants containing the native proteasome complexes are then transferred to fresh safe-lock tubes and are ready for protein concentration assays. Troubleshooting 1
  b. Sample lysis with OK Lysis Buffer is rather easy and only requires ice (DeBruin et al., 2016). After the cell or tissue pellet has been dissolved in OK Lysis Buffer, immediately place the safe-lock tube on ice and continue with the next sample. Once all the samples are dissolved, keep the samples on ice for 20 min incubation time. In between, vortex the samples once and put them back on ice. After 20 min, centrifuge the samples at 4°C for 20 min at 27,670 × *g.* Supernatants are then transferred to new safe-lock tubes and ready for protein concentration assays. Troubleshooting 1

**Figure 1:**
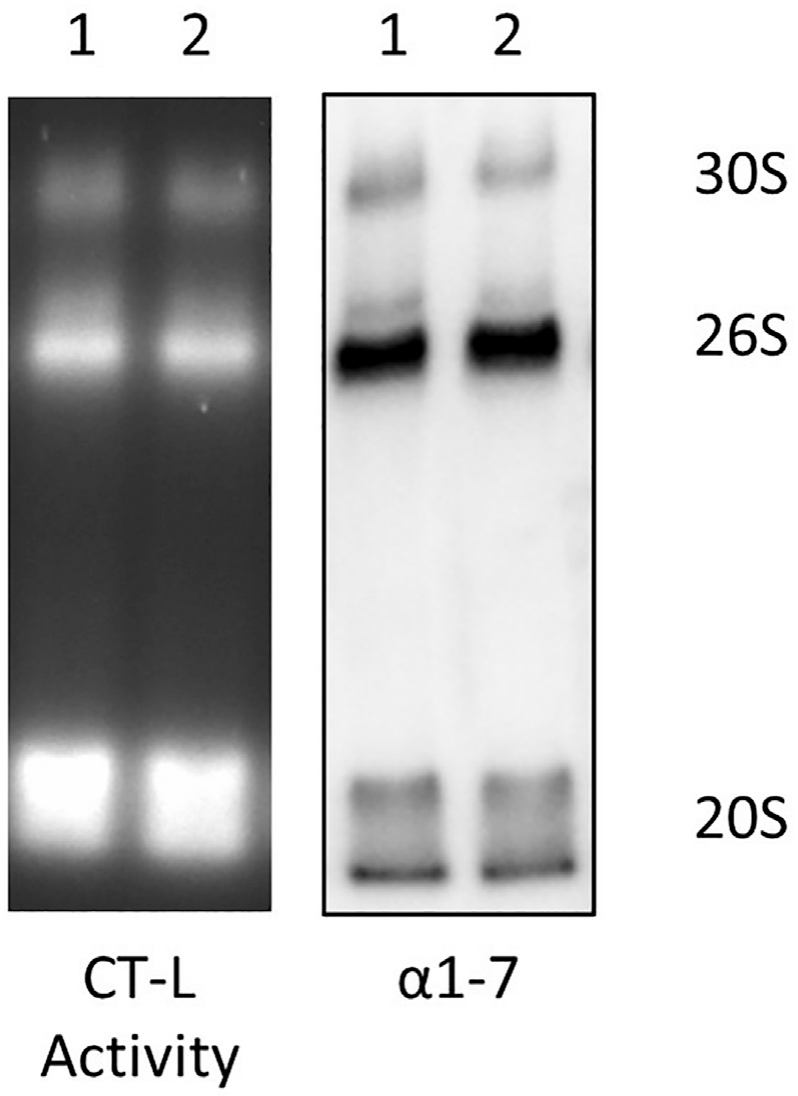
Comparison of OK Lysis Buffer and TSDG Lysis Buffer We used OK Lysis Buffer (1) and TSDG Lysis Buffer (2) conditions to lyse the cells grown under the same conditions and loaded equal amounts of protein onto the gel. The left panel shows the chymotrypsin-like activity of the proteasome complexes, while the right panel shows the immunoblotting of the same gel with α1-7 antibody. There is not apparent difference in proteasome activity and composition when using the two different lysis buffer conditions.
2. Since native gels do not have any proper loading control, you must be very precise in every step of the protocol starting from the determination of the protein concentration to the loading of the gel. We always measure the protein concentration using the bicinchoninic acid assay (BCA assay) and load the same amount of protein from each sample (usually 15 μg) onto the native gel. We use different concentrations of bovine serum albumin (between 0–2 μg/μL) to generate a standard curve. We use 20 μL protein standards, dilute samples 1:10 in PBS, and then add 200 μL BCA reagent according to the manufacturer’s protocol (Thermo Fisher Scientific or other company; https://www.thermofisher.com/order/catalog/product/23225#/23225). After incubation at 37°C for 30 min, we measure absorbance at 562 nm and determine the protein concentration of the sample by comparison of the absorbance with the standard curve.

**CRITICAL:** Additional freezing and thawing of the samples may cause loss of proteasome activity. Therefore, we immediately measure protein concentration after protein lysis and prepare aliquots for subsequent use in native gel assays to avoid additional freeze and thaw cycles of the samples.

## Key resources table

**Table:** 

REAGENT or RESOURCE	SOURCE	IDENTIFIER
**Antibodies**		

Anti-Proteasome 20S alpha 1+2+3+5+6+7 Antibody (1:1000)	Abcam	Cat# ab22674; RRID:AB_2171376
Anti-TBP1 (RPT5) Antibody (1:5000)	Bethyl Laboratories	Cat# A303-538A RRID: AB_10953858
Anti-Proteasome 20S α4 subunit (human), mAb (MCP34) antibody (1:2000)	Enzo Life Sciences	Cat# BML-PW8120 RRID: AB_10541439
Anti-PSMB5 antibody (1:1000)	Abcam	Cat# ab90867 RRID: AB_10675505
Anti-PSMD11 Antibody (1:1000)	Novus Biologicals	Cat# NBP1-46191 RRID: AB_10009423
Anti-PSME1 antibody (1:1000)	Abcam	Cat# ab155091 RRID: AB_2801483
Anti-PA28γ Antibody (1:1000)	Santa Cruz Biotechnology	Cat# sc-136025 RRID: AB_2284426
Anti-Proteasome activator 11S γ subunit antibody (1:2000)	Enzo Life Sciences	Cat# BML-PW8190 RRID: AB_10541407
Anti-PA200 Antibody (1:1000–1:2500)	Novus Biologicals	Cat# NBP2-22236
Anti-mouse IgG HRP-linked Antibody (1:10000–1:40000)	Cell Signaling	Cat# 7076 RRID: AB_330924
Anti-rabbit IgG HRP-linked Antibody (1:10000–1:40000)	Cell Signaling	Cat# 7074 RRID: AB_2099233

**Chemicals, peptides, and recombinant proteins**		

Suc-Leu-Leu-Val-Tyr-AMC	Bachem	I-1395.0100; CAS: 94367-21-2
cOmplete™ Protease Inhibitor	Roche	Ref# 11697498001
Adenosine triphosphate (ATP)	Roche Diagnostics	Ref# BP413-25
Dithiothreitol (DTT)	Life Technologies	Ref# A2948, 0025
Pierce BCA Protein Assay Kit	Thermo Fisher Scientific	Ref# 23225
Immobilon Classico Western HRP substrate	Merck Millipore	Ref# WBLUC0500
Immobilon Forte Western HRP substrate	Merck Millipore	Ref# WBLUF0500

**Software and algorithms**		

Image Lab 6.1	Bio-Rad	https://www.bio-rad.com/de-de/product/image-lab-software?ID=KRE6P5E8Z

**Other**		

NuPAGE 3%–8% Tris-Acetate gel	NOVEX Life Technologies	EA0378BOX
Gel Doc™ EZ System	Bio-Rad	1708270EDU
iBright™ CL1500 Imaging System	Invitrogen	A44114
Balance	Mettler Toledo	XS205

## Materials and equipment

**Table:** TSDG Lysis Buffer

Reagent	Final concentration	Amount
Tris/HCl (100 mM)	10 mM	1 mL
MgCl_2_ (10 mM)	1.1 mM	1.1 mL
NaCl (1 M)	10 mM	100 μL
EDTA (50 mM)	0.1 mM	20 μL
NaN_3_ (100 mM)	1 mM	100 μL
DTT (1 M)	1 mM	10 μL
ATP (200 mM)	2 mM	100 μL
Glycerol (87%)	10% (v/v)	1.15 mL
ddH_2_O	n/a	6.57 mL
**Total**	**n/a**	**10 mL**

To avoid freeze and thaw cycles of the TSDG Lysis Buffer, aliquot it upon preparation into smaller batches and keep it at −20°C until further use. We suggest preparing 10 mL TSDG Lysis Buffer to avoid storing it for longer time. We do not defrost aliquots more than two times, as this might affect the stability of DTT and ATP in the buffer.

**Table:** OK Lysis Buffer

Reagent	Final concentration	Amount
Tris/HCl (pH 7.5, 100 mM)	50 mM	5 mL
DTT (1 M)	2 mM	20 μL
MgCl_2_ (1 M)	5 mM	50 μL
Glycerol (87%)	10% (v/v)	1.15 mL
ATP	2 mM	12 mg
Digitonin (5%)	0.05% (v/v)	100 μL
ddH_2_O	n/a	Add up to 10 mL
**Total**	**n/a**	**10 mL**

To avoid freeze and thaw cycles of the OK Lysis Buffer, aliquot it into smaller batches and keep it at −20°C until further use. We suggest preparing 10 mL OK Lysis Buffer to avoid storing it for longer time. We do not defrost aliquots more than two times, as this might affect the stability of DTT and ATP in the buffer.

**Table:** 8× TBE buffer pH 8.3

Reagent	Final concentration	Amount
Tris Base	712 mM	86. 4 *g*
Boric Acid	712 mM	44 *g*
EDTA-Na_2_	16 mM	5.6 *g*
ddH_2_O	n/a	Add up to 1 L
**Total**	**n/a**	**1 L**

8× TBE Buffer can be stored at 20°C–23°C for 6 months.

**Table:** 5× native gel loading buffer

Reagent	Final concentration	Amount
Bromophenol Blue	0.05% (w/v)	2.5 mg
Glycerol (87%)	43.5%	2.5 mL
Tris (pH 7.5, 500 mM)	250 mM	2.5 mL
**Total**	**n/a**	**5 mL**

**CRITICAL:** The right amount of bromophenol blue in the Native Gel Loading Buffer is very important. Too large amounts increase the viscosity of the loading buffer, which makes it more difficult to load exact volumes of samples onto the gel. Low amounts of bromophenol blue result in reduced coloring and density of the loading buffer making it more difficult to load the sample into the gel pocket. Native Gel Loading Buffer can be stored at −20°C in aliquots. Troubleshooting 2

**Table:** 250× ATP/MgCl_2_

Reagent	Final concentration	Amount
MgCl_2_∗6H_2_O	2.5 M	1.0165 *g*
ATP	250 mM	0.3026 *g*
ddH_2_O	n/a	Add up to 2 mL
**Total**	**n/a**	**2 mL**

**CRITICAL:** 250**×** ATP/MgCl_2_ plays a crucial role in the in-gel proteasome activity assay as ATP stabilizes 26S proteasome complexes. Therefore, we suggest preparing a fresh vial every 1–2 months and store it at −20°C. Older batches might result in reduced activity of the proteasome in the in-gel activity assay due to unstable ATP in the solution. Troubleshooting 3

## Step-by-step method details

### Native page gel electrophoresis

**Timing: 4–5 h**

Native protein extracts are loaded onto native page gels for separation into doubly/singly capped proteasome (30S/26S) and 20S proteasome complexes. 20S Proteasome complexes are usually a mixture of free 20S and 20S capped with one or two proteasome activators (PA28α/β, PA28γ, PA200) depending on the sample.

1. To obtain consistent results between experiments, use NuPAGE 3%–8% Tris-Acetate gel (NOVEX Life Technologies, EA0378BOX). Use 1.5 mm thick gels to increase the amount of loading volume, if necessary.
2. Prepare the samples so that each sample contains exactly 15 μg protein diluted in ddH_2_O with 1× Native Gel Loading Buffer on ice.

**CRITICAL:** Total volume of sample mix should not exceed 20 μL. Increasing the total volume might cause leakage from one gel-loading pocket into the neighboring pocket upon loading.

3. Once the samples are ready, prepare Native Gel Running Buffer.
4. Place the gels into the tank and fill up the gasket with Native Gel Running Buffer (see recipe below) and load samples carefully into the wells.

***Note:*** Do not forget to remove the white strip from the purchased gels before placing it into the tank.

**Table:** Native gel running buffer

Reagent	Final concentration	Amount
TBE Buffer (8×)	1×	100 mL
ATP	413 μM	200 mg
MgCl_2_ (1 M)	2 mM	1.6 mL
DTT (1 M)	0.5 mM	400 μL
ddH_2_O	n/a	Add up to 800 mL
**Total**	**n/a**	**800 mL**

**CRITICAL:** Running Buffer always needs to be prepared freshly and used only once. Do not store Native Gel Running Buffer and do not use it again.

5. After all the samples are loaded, carefully pour the remaining Native Gel Running Buffer into the tank but avoid filling it into the gel pockets. Run the gel at 150 V for 4 h at 4°C (cold room or fridge).

**CRITICAL:** From our experience, the first and last pockets of the gels show different resolution and activity of proteasome complexes in the purchased gels. For this reason, we always use dummy samples (DS), which we load into the first and the last wells of the gel as a balance and avoid leaving empty pockets in the gel. We prepare the dummy samples from cell pellets where we can have a large amount of proteins either using TSDG or OK lysis buffer. For these dummy samples the cell type does not matter. The important part of the dummy sample is that it must contain the same amount of protein, water and native gel loading buffer as the samples to analyze within the same final volume. Since normally we load 15 μg of protein in a pocket with 20-μL final volume, we generally stock dummy samples in PCR tubes which contains 30 μg of protein in 32 μL of water and freeze at −20°C until further use. When we need the dummy samples, we defrost dummy samples and add the 5**×** native gel loading buffer (8 μL). We then load 20 μL of dummy sample to the both ends of the gel. If you have 15 well gel, but only have 10 samples (S), load dummy samples to the remaining 5 wells. An example of loading is given below. Troubleshooting 4

**Table:** 

DS	DS	S#1	S#2	S#3	S#4	S#5	S#6	S#7	S#8	S#9	S#10	DS	DS	DS

### In-gel proteasome activity assay

**Timing: 45 min**

The activity of different proteasome complexes can be measured via in-gel proteasome activity assay. Our protocol is optimized for the analysis of the chymotrypsin-like activity of the proteasome.

6. Defrost 2 mM Suc-LLVY-AMC stock solution one hour prior to the end of running of the gel. Keep it at 20°C – 23°C and in the dark.
7. Prepare Reaction Buffer once the running is finished.

**Table:** Reaction buffer for in-gel proteasome activity assay

Reagent	Final concentration	Amount
Tris (pH 7.5, 100 mM)	1×	12.5 mL
ATP/ MgCl_2_ (250×)	1×	100 μL
DTT (1 M)	1 mM	25 μL
Suc-LLVY-AMC (2 mM, dissolved in DMSO)	48 μM	600 μL
ddH_2_O	n/a	11.775 mL
**Total**	**n/a**	**25 mL**

**CRITICAL:** Reaction buffer always needs to be prepared freshly and used only once. Do not store reaction buffer and do not use it again.

8. Place the Reaction Buffer in a black box to shield it from light and transfer the gel (which has been removed from plates) into the box. Make sure that the Reaction Buffer covers the gel.
9. Incubate the gel for 30 min with the Reaction Buffer at 37°C in the dark box.
10. After incubation, transfer the gel to the plastic holder of the imaging system (e.g., Gel Doc EZ (Bio-Rad) or iBright™ CL1500 Imaging System (Invitrogen)) and image at an excitation wavelength of 380 nm and emission wavelength of 460 nm.

***Note:*** After imaging, transfer the gel immediately back into the box which contains Reaction Buffer. Be careful to not let the gel dry out!

### Immunoblotting

**Timing: 2 days**

Immunoblotting and staining with specific antibodies directed against proteasome subunits or regulators can be used to quantify the amount of active proteasome complexes. Thereby, the composition of the proteasome complexes can be specified regarding regulators attached or catalytic subunits (such as standard or immunoproteasome) incorporated.

11. After measuring the proteasome activity, remove the Reaction Buffer and soak the gel in Solubilization Buffer for 10–15 min at 20°C–23°C.

**Table:** Solubilization buffer

Reagent	Final concentration	Amount
SDS	2% (w/v)	10 *g*
Na_2_CO_3_	66 mM	3.5 *g*
β-Mercaptoethanol	1.5% (v/v)	7.5 mL
ddH_2_O	n/a	Add up to 500 mL
**Total**	**n/a**	**500 mL**

**CRITICAL:** Prepare Solubilization Buffer as a stock solution containing only the first two ingredients, i.e., SDS and Na_2_CO_3_ and store at 20°C–23°C. Add β-Mercaptoethanol (1.5% (v/v)) always freshly once you need the Solubilization Buffer for the experiment. Solubilization Buffer can be used 2–3 times, but we recommend preparing fresh Solubilization Buffer each time. The Solubilization Buffer is important to denature the proteins in the gel. Troubleshooting 5

12. Prepare 1 L of Transfer Buffer (1× transfer buffer, 10% methanol (v/v)).
13. Activate polyvinylidene fluoride (PVDF) membrane (Bio-Rad) in pure methanol for 2 min.
14. Cut off the wells and use the lower thick part of the gel to transfer it to the membrane for immunoblotting using the traditional wet electroblotting method at a constant current of 250 mA for 90 min or 40 mA for 16 hours.
15. Block unspecific binding sites of the PVDF membrane with Roti®-Block solution (Carl Roth) at least for one hour.
16. Add primary antibody diluted in Roti-Block solution either 16–24 h at 4°C or for 1–2 h at 20°C–23°C.

***Note:*** The latter needs fresh primary antibody dilutions.

17. After incubation with primary antibody, wash the membrane three times with PBST (1× Phosphate-Buffered Saline, 1% Tween 20 v/v) for 10 min at 20°C–23°C.
18. Incubate with horseradish peroxidase conjugated secondary antibody diluted to a range of 1:20 000 - 1:40 000 in PBST for 60 min at 20°C–23°C on a shaker.

***Note:*** The dilution for secondary antibody can be adjusted according to primary antibody.

19. After incubation with horseradish peroxidase conjugated secondary antibody, wash the membrane three times with PBST for approximately 30 min at 20°C–23°C.
20. Detect the proteins using LuminataTM Classico or Forte reagent (Merck Millipore) according to manufacturer’s instructions (https://www.merckmillipore.com/) at 20°C–23°C.

**Figure 2:**
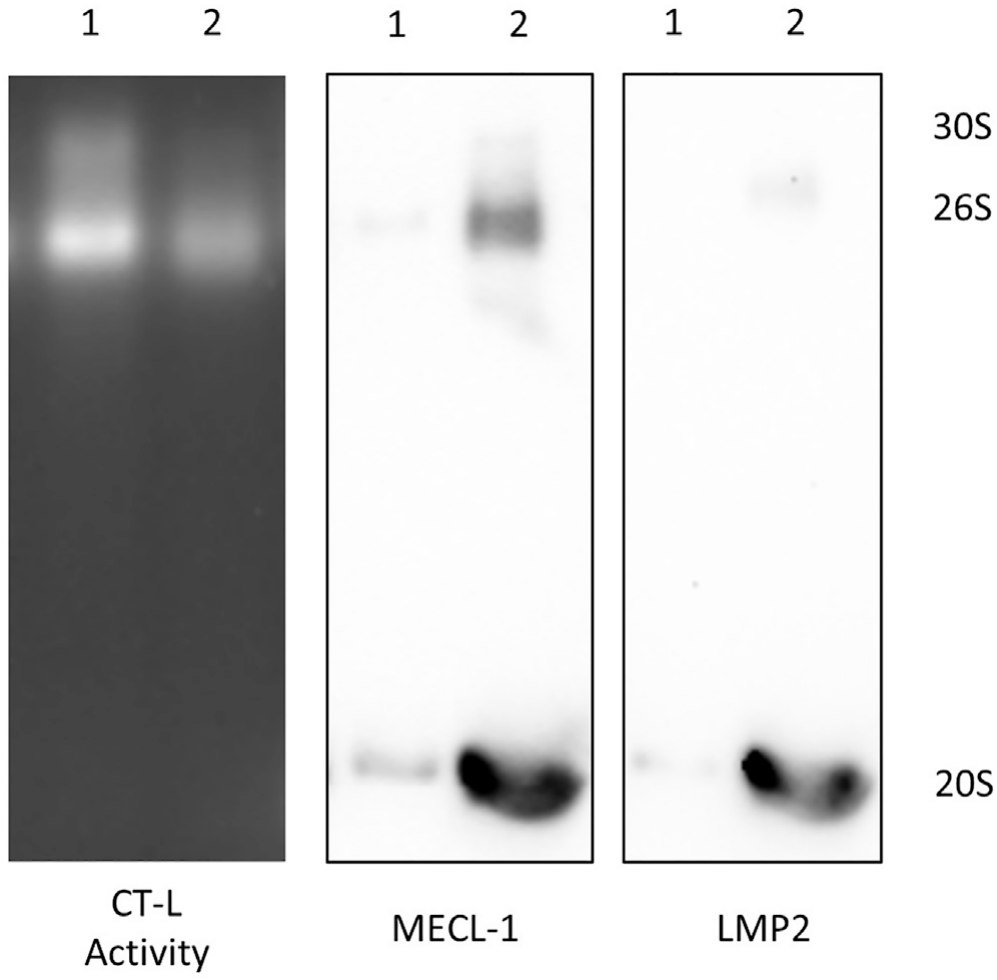
Re-using same membrane for probing with two different primary antibodies A549 cells were treated with IFNγ+TNFα (10 ng/mL and 20 ng/mL, respectively, for 24 h). The first lane (1) contains native lysates from control cells, the second lane (2) contains lysates prepared from A549 cells treated with inflammatory cytokines. After the in-gel proteasome activity assay (left panel), the gel was immunoblotted and incubated with anti-MECL-1 antibody (middle panel). The same membrane was used for detection of LMP2, a second catalytic subunit of the immunoproteasome (right panel). Since both antibodies were from the same species (rabbit) the membrane was incubated with the secondary antibody after stripping/blocking and imaged. The anti-LMP2 antibody was added to the membrane upon confirmation that MECL-1 was not detectable anymore.

**CRITICAL:** If you have enough protein lysate, we encourage you to incubate each membrane with only one antibody. However, note that the membrane can be stripped, blocked and re-incubated with another antibody. Since there is no separation regarding to the size of the subunits, you will possibly detect two different proteins in the same location on the gel corresponding to their integration into 30S, 26S or 20S proteasome complexes. For this reason, make sure that the stripping works. First, strip the membrane and block the unspecific bindings. If your primary antibodies have been generated in the same species (e.g., rabbit-rabbit), incubate your membrane with the secondary antibody directed against this species (e.g., anti-rabbit) after blocking and image the blot (steps 18–20). If you do not get any signal, the stripping of the blot has worked, and you can proceed with adding the other primary antibody (Figure 2). If your primary antibodies are generated in different species (i.e., mouse-rabbit) you can directly proceed to step 20 after stripping/blocking the membrane. If no bands appear, you can add the other primary antibody. If you detect some signals after stripping/blocking procedures, do not use this membrane again for staining with another primary antibody.

## Expected outcomes

In general, you can detect up to three major bands, both in the activity assay and upon immunoblotting. The upper two bands show the 30S, i.e., the doubly capped 20S proteasome, and the 26S proteasome (singly capped 20S) and the lower band shows a combination of different 20S proteasome complexes. Both TSDG and OK Lysis Buffers can be used as non-denaturing lysis buffers without any difference in activity (Figure 1).

**Figure 3:**
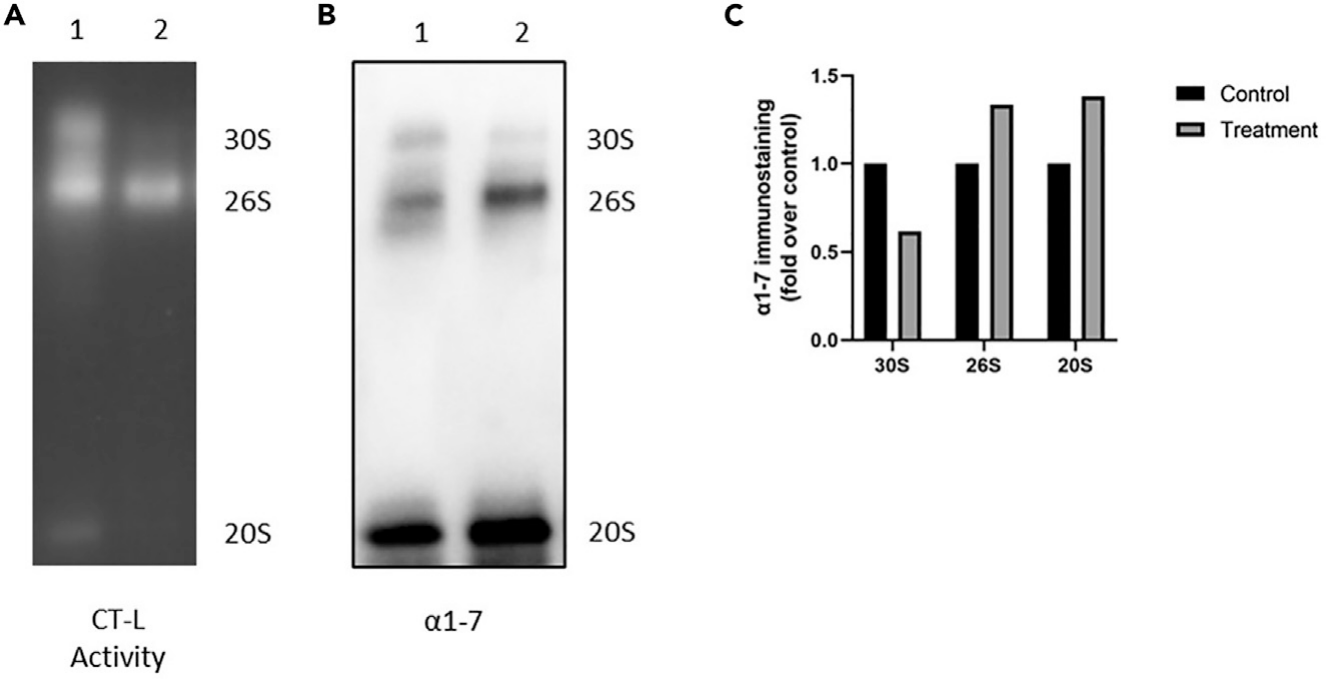
Changes in the activity, amount, and composition of proteasome complexes upon treatment A549 cells were treated with IFNγ+TNFα (10 ng/mL and 20 ng/mL, respectively, 24 h). The first lane (1) contains native lysates from control cells, the second lane (2) contains lysates prepared from A549 cells treated with inflammatory cytokines. (A–C) (A) In-gel proteasome activity assay, (B) Immunoblotting of the same native gel and probing of the membrane with anti-α1-7 antibody, (C) Quantification of α1-7 levels in different proteasome complexes (30S, 26S and 20S).

Upon treatment, the activity, amount and composition of proteasome complexes might change. In this case, you observe different levels of activities and complexes formed. In Figure 3A, the activity of 30S and 20S is reduced upon treatment of A549 lung adenocarcinoma cells with inflammatory cytokines (IFNγ+TNFα). When the gel was immunoblotted and probed with an anti-α1-7 antibody, we detected less 30S but more 26S and 20S complexes upon treatment (Figure 3B). This indicates that the inflammatory treatment either disassembled 30S complexes or resulted in increased assembly of singly capped 26S proteasome complexes and 20S complexes but less assembly of 30S proteasomes. The 20S complexes, however, had less chymotrypsin-like activity. By comparing the activity and amount of proteasome complexes from the same samples of the gel and blot, you can determine the *specific proteasome complex activity* in your samples (i.e., proteasome complex activity/proteasome complex abundance, Figure 3C). You can also determine the ratio between 26S and 20S proteasome complexes. Some of the singly capped 26S and 20S complexes might contain additional proteasome regulators (Wang, Meul and Meiners, 2020) which can be detected by probing with specific antibodies against these regulators (see key resource table for more details). This will then provide you with more information about the composition of proteasome complexes in your sample as for examples shown by Welk et al., (Welk et al., 2016).

## Quantification and statistical analysis

We quantify both the activity and the signals of the immunoblots of native gels. We use Image Lab software to analyze the band intensities (e.g., of the immunoblot in Figure 3B). In order to do this analysis, we define lanes and bands via the “Lane and Bands” tab in the software. We then analyze the band by clicking the “Analysis Table” in the software and copy the analysis table generated by the program (Table 1) to an Excel sheet. The program automatically subtracts background, and the corrected value is given in the Adj. Volume (Int) column. Therefore, we use these values to normalize our data.

**Table 1:** Quantification of immunoblots of native gels, as used in Figure 3B

Lane	Band No.	Band label	Mol. Wt. (KDa)	Relative front	Adj. volume (Int)	Volume (Int)	Abs. quant.	Rel. quant.	Band %	Lane %
**1**	**1**	30S (Control)	N/A	0.14881	2015172	2571291	N/A	N/A	10.52189	3.957284
**1**	**2**	26S (Control)	N/A	0.205357	5222826	5961843	N/A	N/A	27.27014	10.2563
**1**	**3**	20S (Control)	N/A	0.5	11914182	12954654	N/A	N/A	62.20797	23.39642
**2**	**1**	30S (Treatment)	N/A	0.151786	1240542	1870884	N/A	N/A	5.022063	2.204661
**2**	**2**	26S (Treatment)	N/A	0.199405	6978366	7735419	N/A	N/A	28.25039	12.40178
**2**	**3**	20S (Treatment)	N/A	0.497024	16482933	17678250	N/A	N/A	66.72755	29.29307

Upon quantification of activity and amount of proteasome complexes, you can determine the *specific activity* as outlined above as well as the *ratio of complexes*. The ratio will give you information about any alterations in proteasome complex assembly or disassembly upon treatment.

Since native gels do not have proper loading controls such as housekeeping genes, we normalize our samples to the biological controls which we run on the same gel. As an example, we normalized the signal intensities of our distinct proteasome complexes obtained from treated cells (Figure 3B, Lane 2) to the signal intensities of non-treated controls (Figure 3B, Lane 1), respectively. The first and second bands correspond to the 30S complex and 26S complex, respectively. The third band is the core particle of the proteasome, the 20S. Figure 3C shows the α1-7 immunostaining (fold over control), for each proteasome complex.

## Limitations

Although in-gel proteasome activity is a powerful method to measure the chymotrypsin-like activities of different proteasome complexes (such as 30S, 26S and 20S) separately, it is a challenge to quantify trypsin-like and caspase-like activities of the proteasome. In theory, these two activities can be measured with fluorescently labeled substrates like the chymotrypsin-like activity. However, the protocol requires further optimization to obtain reliable results.

We successfully generated robust and reproducible results for human and murine cells. We also tried this method with frozen tissues. Although the fluorescent signals are not as bright as with cell lines, the results can be used for quantifying the activity and composition of the proteasome complexes in tissue (Keller et al., 2015; Semren et al., 2015; Welk et al., 2019). We suspect that the weak fluorescent signals might be due to ineffective native tissue lysis which might be improved by proteasome enrichment by centrifugation and/or generating cell suspensions from tissue.

## Troubleshooting

### Problem 1

No activity in the in-gel activity assay (step: Native Protein Extraction, 1. a and 1. b).

### Potential solution

Make sure that the samples are fresh, and they are not defrosted multiple times. Defrosting of the samples causes loss of activity of the proteasome complexes.

### Problem 2

Formation of smears in the in-gel activity assay (step: 5× Native Gel Loading Buffer Preparation).

### Potential solution

The amount of bromophenol blue in the native gel loading buffer might be too high. Prepare a fresh native gel loading buffer accordingly.

### Problem 3

Loss of fluorescence in the in-gel activity assay (step: 250× ATP/MgCl_2_ Preparation).

### Potential solution

Prepare fresh ATP/ MgCl_2_ (250×). If this solution is old, you may have less fluorescence in the activity assay. Incubate the same gel with new Reaction Buffer containing a fresh batch of ATP/ MgCl_2_ (250×) and you should get better fluorescence intensities.

### Problem 4

The intensity of the first and the last bands are always weak in the in-gel proteasome activity assay (step 5).

### Potential solution

Make sure to use proper number of dummy samples as described. Avoid leaving empty pockets in your gel. If you have 15 well gels and only have 8 samples, fill the other 7 pockets with dummy sample. Keep in mind to load your dummy samples to the outer pockets to stabilize protein separation by gel electrophoresis.

### Problem 5

No bands in the membrane after transfer (step 11).

### Potential solution

Prepare Solubilization Buffer freshly (add β-Mercaptoethanol when you need the buffer) to avoid such problem. Solubilization Buffer is important for the denaturation of the samples in the gel.

## Resource availability

### Lead contact

Further information and requests for resources and reagents should be directed to and will be fulfilled by the lead contact Silke Meiners (silke.meiners@helmholtz-muenchen.de).

### Materials availability

This study did not generate new unique reagents.

### Data and code availability

This study did not generate new data.

## References

[bib1] DeBruin G., Xin B.T., Kraus M., van der Stelt M., van der Marel G.A., Kisselev A.F., Driessen C., Florea B.I., Overkleeft H.S. (2016). A set of activity-based probes to visualize human (immuno)proteasome activities. Angew. Chem. Int. Ed..

[bib2] Keller I.E., Vosyka O., Takenaka S., Kloß A., Dahlmann B., Willems L.I., Verdoes M., Overkleeft H.S., Marcos E., Adnot S. (2015). Regulation of immunoproteasome function in the lung. Sci. Rep..

[bib3] Meul T., Berschneider K., Schmitt S., Mayr C.H., Mattner L.F., Schiller H.B., Yazgili A.S., Wang X., Lukas C., Schlesser C. (2020). Mitochondrial regulation of the 26S proteasome. Cell Rep..

[bib4] Semren N., Welk V., Korfei M., Keller I.E., Fernandez I.E., Adler H., Günther A., Eickelberg O., Meiners S. (2015). Regulation of 26S proteasome activity in pulmonary fibrosis. Am. J. Respir. Crit. Care Med..

[bib5] Wang X., Meul T., Meiners S. (2020). Exploring the proteasome system: A novel concept of proteasome inhibition and regulation. Pharmacol. Therapeut..

[bib6] Welk V., Coux O., Kleene V., Abeza C., Trümbach D., Eickelberg O., Meiners S. (2016). Inhibition of proteasome activity induces formation of alternative proteasome complexes. J. Biol. Chem..

[bib7] Welk V., Meul T., Lukas C., Kammerl I.E., Mulay S.R., Schamberger A.C., Semren N., Fernandez I.E., Anders H.J., Günther A. (2019). Proteasome activator PA200 regulates myofibroblast differentiation. Sci. Rep..

